# OSCE best practice guidelines—applicability for nursing simulations

**DOI:** 10.1186/s41077-016-0014-1

**Published:** 2016-04-02

**Authors:** Michelle A. Kelly, Marion L. Mitchell, Amanda Henderson, Carol A. Jeffrey, Michele Groves, Duncan D. Nulty, Pauline Glover, Sabina Knight

**Affiliations:** 1 Curtin University, Kent Street Bentley, Perth, Western Australia 6102 Australia; 2 Griffith University and Princess Alexandra Hospital, University Drive, Meadowbrook, Queensland 4122 Australia; 3 Princess Alexandra Hospital and Griffith University, 199 Ipswich Rd, Woolloongabba, Queensland 4102 Australia; 4 University of Queensland, St Lucia, Queensland 4072 Australia; 5 Australian Catholic University, Level 21, Tenison Woods House, 8-20 Napier Street, North Sydney, NSW 2060 Australia; 6 Flinders University, Sturt Rd, Adelaide, 5042 South Australia Australia; 7 James Cook University, 1 James Cook Drive, Townsville, Queensland 4811 Australia

**Keywords:** OSCE, Simulation, Best practice guidelines, Pedagogy, Holistic practice, Affective domain

## Abstract

**Background:**

Objective structured clinical examinations (OSCEs) have been used for many years within healthcare programmes as a measure of students’ and clinicians’ clinical performance. OSCEs are a form of simulation and are often summative but may be formative. This educational approach requires robust design based on sound pedagogy to assure practice and assessment of holistic nursing care. As part of a project testing seven OSCE best practice guidelines (BPGs) across three sites, the BPGs were applied to an existing simulation activity. The aim of this study was to determine the applicability and value of the OSCE BPGs in an existing formative simulation.

**Methods:**

A mixed methods approach was used to address the research question: in what ways do OSCE BPGs align with simulations. The BPGs were aligned and compared with all aspects of an existing simulation activity offered to first-year nursing students at a large city-based university, prior to their first clinical placement in an Australian healthcare setting. Survey questions, comprised of Likert scales and free-text responses, used at other sites were slightly modified for reference to simulation. Students’ opinions about the refined simulation activity were collected via electronic survey immediately following the simulation and from focus groups. Template analysis, using the BPGs as existing or a priori thematic codes, enabled interpretation and illumination of the data from both sources.

**Results:**

Few changes were made to the existing simulation plan and format. Students’ responses from surveys (*n* = 367) and four focus groups indicated that all seven BPGs were applicable for simulations in guiding their learning, particularly in the affective domain, and assisting their perceived needs in preparing for upcoming clinical practice.

**Discussion:**

Similarities were found in the intent of simulation and OSCEs informed by the BPGs to enable feedback to students about holistic practice across affective, cognitive and psychomotor domains. The similarities in this study are consistent with findings from exploring the applicability of the BPGs for OSCEs in other nursing education settings, contexts, universities and jurisdictions. The BPGs also aligned with other frameworks and standards often used to develop and deliver simulations.

**Conclusions:**

Findings from this study provide further evidence of the applicability of the seven OSCE BPGs to inform the development and delivery of, in this context, simulation activities for nurses. The manner in which simulation is offered to large cohorts requires further consideration to meet students’ needs in rehearsing the registered nurse role.

**Electronic supplementary material:**

The online version of this article (doi:10.1186/s41077-016-0014-1) contains supplementary material, which is available to authorized users.

## Background

Preparation of healthcare students for clinical practice experiences has long been an important yet challenging area of education programmes [1, 2]. A range of teaching and assessment strategies have been used to assist with this aspect of curricula. Objective structured clinical examinations (OSCEs) have been used for decades within nursing and medical programmes to assist with preparation for practice, scaffold learning, determine participants’ level of clinical performance and provide feedback on areas for improvement [3–6]. OSCEs generally feature a number of skills stations (typically 8–10 with 5–8 min allowed per station) which students rotate through to test discrete knowledge and clinical and professional skills [5]. Objectivity of the assessment is achieved by assessors using rating scales or checklists to make judgements of mandatory competencies through observing students’ performances [7]. Variability in the development, delivery and quality of the processes fundamental to the OSCE have been identified as problematic for ensuring consistent value of the learning experience for students [8–10]. Further, attention to the affective domains of practice, central to holistic patient care, are not always acknowledged or captured within assessments of clinical competence which often focus on acquisition of *technical* skills [11, 12].

For OSCEs to be effective in student preparation for holistic practice, they need to assess more than the technical skills, particularly the affective elements of practice, and be grounded within and informed by educationally sound principles [13]. Following literature review and evaluative work, OSCE best practice guidelines (BPGs) were developed, successfully trialled and tested to provide an evidence-based approach to guide academics in maximising the benefits of this educational strategy [14]. The BPGs incorporate all elements of this complex activity such as content (focusing on safe patient care), holistic marking guide, mastery of skills, sequencing (including briefing), supportive environment, feedback and ongoing practice for both formative and summative assessment (see Table 1, left column).

**Table 1:** OSCE BPGs [14] and modifications made for the SIM activity, with examples

Best practice guideline	Modifications to the SIM activity
1. Focuses on aspects of practice related directly to the delivery of safe patient-centred care	No change.
2. Includes practices which are most likely to be commonly and/or significantly encountered	No change.
3. Will be judged via holistic marking guide to enhance both the rigour of assessment and reliability Allows for judgement of students’ performance as a whole rather discrete independent actions	As the simulation was a formative learning activity, there was no marking guide. However, debriefing strategies and trigger questions were used to connect performance with clinical practice.
4. Requires students to perform tasks in an integrated rather than piecemeal fashion by combining assessments of discrete skills in an authentic manner	No change.
5. Will be structured and delivered in a manner which aligns directly with mastery of desired knowledge and skill	No change.
6. Will be appropriately timed in the sequence of students’ learning to maximise assimilation and synthesis of disparate course content and to minimise the potential for students to adopt a piecemeal, superficial learning approach	No change.
7. Allows for ongoing practice of integrated clinical assessment and intervention skills in a secure supportive environment thereby ensuring appropriate provision of feedback to guide students’ development	Adequate opportunities for continued practice with feedback were included during or soon after each clinical tutorial class and during the debrief. However, academics were made more aware of this aspect leading up to the SIM.

A particular focus of the BPGs is for students to appreciate an integrated approach to patient care (building a therapeutic nurse-patient relationship and individualising care) rather than solely focusing on correct skill performance. In addition, the use of a global rating scale, particularly when assessment is formative, offers greater context and meaning during feedback compared with a checklist format [12]. Building on previous work [14], this current project extended inquiry about the applicability of the BPGs for different populations at varied sites [15, 16] resulting in refinement of the seven BPGs (Table 1) [17]. Further inquiry, which we report in this paper, focused on ways in which the OSCE BPGs may align with healthcare simulations.

One type of contemporary healthcare simulations (SIM), often used to rehearse clinical scenarios and timed to prepare students for practice, has seen significant growth over the last 15 years [18–20]. SIMs frequently incorporate two or more participants from the same or multiple health disciplines who progress through a clinical scenario, respond to changes in a 'patient's' condition, and then discuss outcomes through a facilitated debriefing [18, 21]. If prepared and delivered appropriately, simulations can approximate actual clinical practice and inform participants’ learning particularly through the debriefing process [22]. Although early frameworks for SIM activities were available at the time of this study [18, 21], we were interested in determining if the BPGs could provide an additional perspective given the similarities between OSCEs and SIMs. The research question was in what ways do OSCE BPGs align with simulation?

## Methods

A mixed methods approach was used to explore the applicability of the OSCE BPGs for SIMs. The applicability of the BPGs was explored by examining and ensuring alignment to an existing SIM for first-year nursing students in a mandatory subject where the SIM was timed prior to the first clinical experience. Applicability was informed by data from (1) academics’ perceptions of applicability of the guidelines, (2) student survey and (3) student focus groups to explore the impact of the newly aligned SIM activity on learning.

### Alignment of OSCE BPGs to an established SIM activity

Planning for the SIM activity included a site visit by members of the research team (MM and CJ). With the local SIM expert (MK) and the subject coordinator, the intent was to compare and refine the existing SIM with the OSCE BPGs in relation to the teaching, delivery and assessment processes. This exercise demonstrated that the majority of the guidelines were relevant, already in use, and contributed to the cogency of the revised SIM at this site. Table 1 outlines the relevant modifications made for the SIM at this site (*right column*) in relation to the OSCE BPGs.

### The SIM activity

The SIM activity was scheduled in the third quarter of a 12-week semester and comprised of three parts—rehearsal, SIM and debriefing (Table 2). Theoretical and practical content informed the patient case scenarios used in the SIM rehearsal. Students had opportunity to practice related skills in clinical skills laboratories (during classes and in free time) in the 4 weeks leading up to the SIM activity. In total, the SIM activity lasted 3 h. The students were allocated into groups of twelve. During the rehearsal, the students worked in triads through similar patient case scenarios to refresh clinical skills likely to be included in the SIM. When ready to move into the SIM, three students participated in the first scenario, three other students took on roles in the second scenario while the remaining six students participated in the third scenario. Further details including student preparation are provided in Table 2. This *cycle* could be repeated up to five times in 1 day and when doubling the number of laboratories, a total of 120 students could be offered the SIM activity per day. Over 5 days, 600 students could be accommodated.

**Table 2:** Brief description of the three-part SIM activity

1. Rehearsal—90 min
Students refreshed the skills likely to be required in the SIM. In groups of three, students rehearsed techniques in the context of two patient case studies with guidance provided by a clinical facilitator^a^. Immediately prior to the end of part 1, students watched a short video of the SIM activity with roles played by clinicians who modelled holistic practice.
2. SIM—45 min
There were three SIM scenarios facilitated by academics. Students actively participated in a role in one scenario then observed peers enacting roles in other scenarios.
Students were required to exhibit communication techniques through a patient education scenario. Roles included the following: an anxious mother, an adolescent son who was not engaged with his asthma management and a practice nurse. This scenario was run twice requiring three students to demonstrate an interaction with less effective communication and then another three students to demonstrate effective communication techniques.
The third scenario allowed the other six students to engage in roles. The scenario was an elderly male patient (manikin) experiencing chest pain who then deteriorated and required CPR. The patient’s voice was allocated to one student (in the control room); another two students enacted the roles of wife and daughter; and the remaining three students (in nursing roles) provided CPR, initiated assistance by telephone and interacted with the family to explain the situation.
3. Debriefing—45 min
Facilitated debriefing by the academics occurred in a number of ways: for example, a short debrief occurred immediately following each communication scenario, then a longer debrief occurred after the final CPR scenario. Time was available during the longer debrief to revisit and discuss points of interest or contention from any of the three scenarios. The debrief was structured using *pre-determined trigger questions* to elicit students’ thoughts about what was observed, overall performance and responses to the situations, teamwork, communication and clinical decision making.

^a^Clinical facilitator is a clinical nurse, contracted by the university, to oversee students during clinical practice experiences in the service sector

In preparation for the SIM activity, the relevant academics executed their own rehearsal of the scenarios. This helped them to understand the intent of the activities and to gain insight to how students would feel participating in the scenarios, offering greater appreciation of the facilitation processes required. The local SIM expert (MK) and subject coordinator directed and audio-visually (AV) recorded the staff rehearsal and modelled debriefing practices to improve consistency in this particular area across the staff cohort. Detailed SIM guides were prepared for staff to inform all aspects of the SIM activity. An edited version of the AV recording was shown to the students at the end of stage 1 to provide them with a schema of what SIM may be and to demonstrate professional nursing behaviours.

### Evaluation

The revised SIM activity (see Tables 1 and 2), was evaluated by the study participants. Immediately following the SIM activity, the students completed an online survey.

The survey consisted of 17 items to obtain student feedback on the three-part SIM activity (see Additional file 1: Appendix 1). The majority of questions required fixed responses (seven-point Likert scale) and one item allowed for free-text comments. Six of the questions required two-part responses (for example, just getting the skills right and using an integrated approach). The survey had been piloted with student groups at two other sites [15, 16]. All students had ready access to computers to complete survey questions.

In addition to the online survey, four student focus groups were conducted 1 week after the SIM activity. Each group comprised of up to 12 students. For each group, one (external) researcher (MM) guided discussions (see Additional file 2: Appendix 2) to elicit further feedback on the utility of the revised SIM activity for student learning and as preparation for practice. The local researcher (MK) took responsibility for writing detailed notes of the students’ responses as preference over audio-recording. Although known to students, the local researcher was not involved directly with teaching or facilitating the SIM activity, was positioned outside the group *and* did not participate in discussions. Notes from each focus group were handed to the facilitator (MM) who checked with participants to ensure an accurate account of the dialogue and conversations was captured [23]. This was the most convenient approach as it allowed for immediate check back of concepts with all students.

### Ethical considerations

Approval for the study was obtained from the university ethics committee (University of Technology Sydney). The students were informed of the study before the SIM activity and that there were no added benefits or course credit from participating in the research. Details of the research preceded the online survey and completion of the survey indicated consent. The students voluntarily chose to participate in focus groups and signed consent forms prior to the commencement of the discussion. To limit bias or influence, the local researcher (MK) and the subject coordinator did not participate in the SIM activity nor lead the focus groups. The SIMs were not audio-visually recorded.

### Participants and site

Research participants were a convenience sample of first-year Bachelor of Nursing students from a large metropolitan university in Australia. As the research was conducted during the first semester of their Bachelor of Nursing degree, the students had minimal or no previous clinical experience within an Australian healthcare setting, although some of the international students had experience in their country of origin. Following an email invitation to all students, 47 agreed to participate in the focus groups.

### Data collection, management and analysis

Numerical data from the online survey were entered into the Statistical Package for Social Sciences (SPSS Version 20) data analysis package. Numerical data were summarised using descriptive statistics.

Focus group data and responses to the open-ended online survey question were analysed by four of the researchers (CJ, MM, MK and AH) using template analysis method where the OSCE BPGs were used as existing or a priori themes [24]. Similar to other techniques of analysing qualitative data, an iterative approach was adopted with each researcher individually reviewing and coding the data, using the BPGs as a template, to determine the impact of the revised SIM activity on student learning. Subsequent comparisons by the four researchers helped to refine evidence to support or challenge the OSCE BPG template and reach agreement on themes. Data were converged to provide a deeper and richer understanding of student feedback and corroborate the results [23, 25].

## Results

Responses were received from only two academics. Both taught in the subject and delivered the SIMs. One had greater experience in simulation development and delivery while the other was a novice in this teaching and learning approach. The academics were pleased that the educational structure and intent of the SIM aligned so closely with the OSCE BPGs. Specific comments referred to SIM as a positive learning strategy which enabled students to “glue” things together (use an integrated approach); although there were ample opportunities for students to practice beforehand, as the SIM drew closer, there was greater motivation by students to rehearse the activities. The staff were undecided at this point about using SIM for summative assessment as is the case in OSCEs.

Responses to the electronic survey totaled 367 from a possible 457 students (80 % response rate). Forty-seven students contributed to one of the four focus groups. The majority profile of students who participated in the post-SIM survey were between 17 and 50 years with a mean age of 23.4 years, female (*n* = 306; 86.2 %), recent school leavers (*n* = 112; 30.6 %) and international students (*n* = 129; 35.1 %) (see Table 3). The study sample represents the nursing student cohort at this university.

**Table 3:** Category, number and percent of students who completed the post-simulation survey

Student category	*N* = 367	Percentage
School Leaver	112	30.6
Mature age/non-recent school leaver	92	25.1
Graduate entry	24	6.5
International	129	35.1
Missing data	10	2.7

### Student experience of the revised SIM activity

The surveys and focus groups provided insights about the applicability of the seven BPGs in the development and delivery of the SIM activity. According to students, the benefits of the three-part SIM activity (informed by the BPG statements) directly related to the teaching, learning and assessment principles which contributed to the breadth of learning. Findings are reported with respect to each of the BPGs to reflect the template analysis approach.

**BPG 1:** Focus on aspects of practice related directly to the delivery of safe patient-centred care; AND

**BPG 2:** Focus on aspects of practice which are most relevant and likely to be commonly encountered

A survey question asked whether students felt the SIM activity provided them with a real-life clinical scenario. The large majority of students (*n* = 341; 93 %) agreed that from their perceptions, the SIM felt like a *real-life* situation thus confirming the relevance of the SIM activity to their understanding of clinical practice. The focus groups further confirmed that the content and context of the scenarios were relevant and helped prepare them for the upcoming direct clinical experience and observation. Two students commented in this regard:

Much more real situation… better than labs, made me feel more comfortable

Focus Group 4

Glad the SIM was there as I felt not prepared enough for clinical practice as I’d done a lot of self-directed learning- too much …needed this (SIM).

Focus Group 2

What emerges here is the contribution of this SIM activity towards students “knowing how” for clinical rather than learning facts, details and procedures in isolation of context or of each component part [26]. Knowing and understanding through combining the relevant aspects of practice and safe patient-centred care within the scenarios led to a positive attitude for the *real* situation.

**BPG 3:** Be judged via holistic marking guide to enhance both the rigor of assessment and reliability. This allows judgement of students’ performance to be related to clinical practice as a whole rather than as a collection of discrete independent actions.

In relation to BPG 3, while this SIM activity did not involve a summative assessment, the format of the debriefing provided a proxy holistic guide (for discussion and feedback) akin to an OSCE marking guide. Overall, students positively rated the feedback received on their performance during preparation for the SIM activity and during the post-SIM debrief. Ninety percent of the students (*n* = 330) indicated they received feedback from teachers (sometimes, mostly or always), and specifically 77 % (*n* = 284) thought the feedback was helpful or very helpful (Tables 4 and 5). Both preparation for and participation in the SIM activity provided additional opportunities for informal peer feedback (93 % *n* = 341) which in 71 % of cases (*n* = 260) was rated as helpful or very helpful (Tables 4 and 5).

**Table 4:** Student responses (*n* and %) to survey questions about availability and helpfulness about feedback

Question	Number	Not at all	Sometimes	Mostly	Always
Did you receive feedback from teaching staff when practising for the simulation?	315	32 (10.1 %)	*120 (38.1 %)*	*106 (33.7 %)*	57 (18.1 %)
Did you receive feedback from peers when practising for the simulation?	312	21 (6.7 %)	*145 (46.5 %)*	*99 (31.7 %)*	47 (15.1 %)

Values in italic represent the two highest rating categories for each question

**Table 5:** Student responses (*n* and %) to survey questions about availability and helpfulness about feedback

Question	Number	Very unhelpful	Unhelpful	Slightly unhelpful	Undecided	Slightly helpful	Helpful	Very helpful	N/A
How helpful was the feedback from the teaching staff when practising the simulation?	314	8 (2.5 %)	3 (1 %)	4 (1.3 %)	12 (3.8 %)	32 (10.2 %)	*108 (34.4 %)*	*135 (43 %)*	12 (3.8 %)
How helpful was the feedback from peers when practising the simulation?	312	7 (2.2 %)	3 (1 %)	3 (1 %)	15 (4.8 %)	53 (17 %)	*138 (44 %)*	*85 (27 %)*	8 (2.6 %)

Values in italic represent the two highest rating categories for each question

*N/A*, not available

The students also indicated through the focus groups that feedback, during the debrief in this instance, was important in developing their own judgements about performance and clinical practice.

We were able to sit down and say “if I did it again right now” I would change this or that.

Focus Group 1

In debrief you notice things and [were] told things you didn’t even realise you did – good to learn.

Focus Group 3

I think it helped me in a positive way- different teachers and different students (than usual classes) - it was good it was mixed up - learn from other people and their mistakes- bouncing ideas off each other.

Focus Group 3

It appears that the feedback provided, particularly through debriefing, helped the students develop constructive approaches to dealing with situations. However, the students indicated that some of the debriefers had not addressed the issues which were important to them or that discussions were not in-depth or constructive enough when talking about the communication elements of the SIM activity.

**BPG 4**: Require students to perform tasks in an integrated rather than piecemeal fashion by combining assessments of discrete skills in an authentic manner.

For a number of concepts, the students were asked to discern between “just getting the skills right” and “using an integrated approach”. The phrase “just getting the skills right” was explained to the students as *only* focusing on the clinical skills in the SIM activity. Alternatively, “using an integrated approach” enables the students to focus on all the skills required for holistic care such as developing a therapeutic nurse-patient relationship, providing comfort and privacy and individualising patient care.

The students perceived that the SIM activity was more real-life when they focussed their nursing care in an integrated manner for the 'patient's' (90 %, *n* = 330) in contrast to just focussing on the required skills (70 %, *n* = 257) (Table 6).

**Table 6:** Students’ survey responses regarding preparation for simulation

Question	Combined responses *n* (%) for: slightly agree/agree/strongly agree
To do well in the simulation, I thought I would do well enough by:	
a) Just getting the skills right	242 (76)
b) Using an integrated approach	288 (88)
When I practised for the simulation throughout the semester I focused on:	
a) Just getting the skills right	274 (86)
b) Using an integrated approach	265 (83)
The simulation felt more real-life when I focused on:	
a) Just getting the skills right	226 (70)
b) Using an integrated approach	292 (90)
Nearing the time of the simulation, I focused my preparation on:	
a) Just getting the skills right	285 (89)
b) Using an integrated approach	245 (76)

**BPG 5**: Be structured and delivered in a manner which aligns directly with mastery of desired knowledge and skill. This alignment should be both internal to the course and aligned prospectively with clinical tasks likely to be commonly and/or significantly encountered in practice.

**BPG 6:** Be appropriately timed in the sequence of students’ learning to maximise assimilation and synthesis of disparate course content and to minimise the potential for students to adopt a piecemeal, superficial learning approach.

The students indicated that the scenarios were authentic and meaningful to them. As students indicated that the SIM was relevant, it suggests that the SIM activity was appropriately aligned to their subject progression and timed in the sequence of learning. One student highlighted the progressive nature of their learning within the subject (and other students concurred) saying:

(The SIM activity) builds up the level of complexity… it builds the situation up for us

Focus Group 4

SIM has taken the edge off the worry about the anticipation of clinical.

Focus Group 2

The importance placed on actively playing the registered nurse role in the SIMs was heightened through the following survey quote:

The debrief went on too long. I would have liked to do other roles (but I) only got to be the patient

Survey

These comments indicated that while the SIM activity supported students in their preparation for clinical practice, some students indicated less benefit when they were asked to undertake a role other than the nurse.

In summary, the data indicated that an integrated approach to learning was important for students. When they practised throughout the semester, the students responded that they focused on both “just getting the skills right” (86 %) and also “using an integrated approach” (83 %). However, closer to the time of the SIM activity, the students indicated that they focused more on “just getting the skills right” (89 vs 76 %) (Table 6).

General comments from the focus groups that reinforced the value of assimilating clinical knowledge with the SIM activity included the following examples:

I felt that this [SIM] was a huge help to me, it taught me a lot by putting it into practice with my peers and made me think and prepare even more so for clinical.

Focus group 2

…it really helps with your clinical placement, the situation is more realistic than the regular lab.

Focus group 1

It is a very nice way to improve the skills in the student before facing a real life situation so it should be an ongoing process.

Focus group 4

**BPG 7:** Allow for ongoing practice of integrated clinical assessment and intervention skills in a secure supportive environment thereby ensuring the appropriate and provision of feedback to guide students’ development and ongoing reflection.

Eighty percent (*n* = 294) of the students responded that they had adequate practice time during and after clinical lab classes thus indicating that structured preparation for the SIM in the programme was effective in facilitating continuing student engagement with the activity, to illustrate:

I appreciate those teachers who checked our skills and gave us their feedback therefore we have the opportunity to change our errors and do practice in [the] right way.

Survey

The value of the secure environment for the SIM activity was particularly evident through the focus group comments such as:

I very much enjoyed simulation. Having a relaxed, judgement free, small group environment helped me to feel comfortable practising my skills and being able to easily fall into role play without feeling too self-conscious.

Focus group 2

Knowing that it is not an assessment takes the tension off, it enables me to participate and raise questions more freely, and I could really ‘act out’.

Focus group 4

A final student comment drawn from the survey open-ended questions summarised the value of the BPGs upon which the SIM activity was aligned, particularly in relation to timing and using an integrated approach:

Simulations and OSCEs can be confronting and an almost threatening situation and should be held later as not to impose or cause extreme anxiety on a student. In a safe setting where we can make mistakes is good practice and great for the integration of knowledge rather than set skills.

## Discussion

This research explored how OSCE BPGs aligned with SIMs. Allowing for minor differences across both approaches, each BPG was accounted for within the existing SIM activity offered to first-year nursing students. The research provided opportunity to review the processes of planning and delivering SIMs at this university and to revise steps where necessary. Feedback from students and academics verified the benefits of the revised SIM activity. One specific difference between the BPGs and the SIM activity was that a holistic marking guide, recommended for OSCEs, was not used in this formative SIM. However, discussions about clinical performance (often included in a marking guide) were the basis of these post-SIM debriefings.

Variations in the applicability of OSCE BPGs for SIMs may arise depending on the scenario context and experience of participants. The purpose of this SIM activity was to assist nursing students in preparing for their first clinical placement in an Australian healthcare setting. Specifically, the intent was to impress on students a comprehensive approach to patient care to assist the development of students’ affective domains in addition to psychomotor skills [11]. The SIM activity was framed within the context of clinical activities likely to be encountered which required communication and other clinical skills responses from students. SIM can be a powerful experience which assists students to embody practice, moving from *knowing* (facts, requisite knowledge) to *knowing how* (application of or combining and orchestrating numerous processes within clinical practice) which unlike other traditional learning strategies, facilitates understanding of and about practice [26, 27]. This specific SIM format had been developed and delivered on previous occasions [28, 29] and aligned not only with the OSCE BPGs [14] but also with subsequently published quality frameworks and standards for SIM [30–34].

Students’ responses from both the survey and focus groups indicated that BPGs were applicable and workable for SIMs in guiding student development across the affective, cognitive and psychomotor domains that are essential in their preparation for practice. This is congruent with other studies which used the BPGs for OSCEs to support learning for Bachelor of Midwifery students [16] and post-graduate nursing students in remote settings [15].

The appropriateness of the BPGs in this current study suggests that these guidelines provided both a transparent and pedagogically sound direction to the development and implementation of the SIM that assisted in strengthening the quality of students’ learning. Further, the BPGs align with more recently published standards for SIM [31–34] which endorses the applicability of these OSCE BPGs as an additional evidence-based framework for developing and delivering SIMs.

Two key findings regarding the application and use of OSCE BPGs for SIM have emerged from this study and relate to the challenges of large student cohorts. The first finding pertains to the number of students who were able to enact the nurse role. As discussed, each time this activity was undertaken, it was with small groups of students (*n* = 12) with only five of the 12 students able to take on the nurse role. The students, who were cast in a role other than the nurse, indicated in their feedback that the SIM activity did not directly align with the experience they wanted.

The second finding relates to the value of “a holistic marking guide” and the nature of assessment predominantly used in SIM, that is, formative assessment. The variation of responses from the focus groups highlighted that “a holistic debriefing guide” (or in this instance, specific universal pre-determined questions to trigger responses during the debrief) was beneficial as it facilitated the breadth of intended discussion during the debriefing. A particular similarity to BPG 3 is that debriefing assisted students to develop their own judgements about performance and clinical practice. However, students indicated that more equity, in allowing every student to contribute to the debriefing discussions, would benefit all in their learning and reflection; this requires the facilitator to have adequate moderating skills [22, 35, 36].

The use of a more structured debriefing guide and further instruction on how to lead these discussions would be beneficial for staff in clarifying the priorities for student learning [35, 36]. A debriefing guide relevant to these specific scenarios and student level could also enhance the consistency of discussions and the student experience when managing large cohorts. This is a particular challenge as the majority of teaching academics are casual staff with varied experiences of facilitating SIMs, and the SIM activity is time limited by a tight schedule. A refined debriefing guide has already been implemented, and an experienced SIM academic guides and supports new staff during the debrief component of the SIM activity to improve the equity of students’ learning experiences. Further exploration of these two key findings is being considered by the research group.

### Strengths and limitations

Findings are from a single site and one cohort of students. Although the participant numbers for the survey data were relatively large, and numbers within the focus groups aligned with recommended research practice [23], feedback from the focus group participants may have been skewed due to self-selection. The survey was post-experience only; a pre/post-SIM survey may have allowed for comparison of data points. However, the intent of this research was not to disrupt students’ preparation for the SIM but rather to gain their experiences of the revised SIM activity in relation to how the OSCE BPGs were applied overall.

## Conclusions

This paper reports on students’ experiences, perceived outcomes and recommendations regarding the applicability of the OSCE BPGs for a three-part SIM activity for first-year nursing students. Similarities exist between frameworks and standards often used to develop and deliver SIMs and the OSCE BPGs. Findings from this study provide further evidence of the utility of the seven BPGs to inform the development and delivery of SIMs and feedback as preparation for practice across the affective, cognitive and psychomotor domains. This is consistent with findings from two other studies within a larger research project evaluating the BPGs at other sites, with post-graduate nurses in remote settings and student midwives at another metropolitan university [15, 16]. The manner in which OSCE BPGs in SIM is practised with large cohorts requires further consideration to meet students’ needs in rehearsing the registered nurse role.

## Additional files

Additional file 1: Student survey questions. (DOCX 63 kb)

Additional file 2: Examples of questions in students’ focus groups. (DOCX 24 kb)
