# Supplementary material for: OSCE best practice guidelines—applicability for nursing simulations
Source: Adv Simul (Lond). 2016 Apr 2;1:10. doi: 10.1186/s41077-016-0014-1 (PMC5806284; doi:10.1186/s41077-016-0014-1)
Supplement: Supplementary file 1 — Student survey questions. (DOCX 63 kb) [file 41077_2016_14_MOESM1_ESM.docx]

**Appendix 1 – Student survey questions**

**An Implementation framework for Simulation ‘Best Practice Guidelines’ designed to improve nurse preparedness for practice.**

**Please complete the following-**

**My age is:**

________ years

**I am:**

Male ₁ Female ₂

**Please choose one of the following, I am a:**

School Leaver ₁ Mature Age Student ₂ Graduate Entry Student ₃ International Student ₄

Other Please Specify: _____________

**Instructions for Questions 1 to 6:**

**Please answer the following questions by circling the number for each question both Part A and B which you think best represents your thoughts about your simulation performance and preparations.**

**Part A is asking about:**

- **Just getting the skills right-** that is only focusing on the clinical skills in the simulation.

**Part B is asking about:**

- **Using an integrated approach-** that is not only focusing on the clinical skills but also bringing in all aspects of a therapeutic nurse-patient relationship such as comfort and privacy etc.

| **1. The simulation is roughly like a real-life situation. I believed I could do well enough by:** | | | | | | | | |
| --- | --- | --- | --- | --- | --- | --- | --- | --- |
|  | Strongly Disagree | Disagree | Slightly Disagree | Undecided | Slightly Agree | Agree | | Strongly Agree |
| **A)** Just get the skills right | 1 | 2 | 3 | 4 | 5 | 6 | | 7 |
|  | | | | | | | | |
| **B)** Use an integrated approach | 1 | 2 | 3 | 4 | 5 | 6 | 7 | |
| **2. To do well in the simulation I thought I would need to:** | | | | | | | | |
| **A)** Just get the skills right | 1 | 2 | 3 | 4 | 5 | 6 | 7 | |
|  | | | | | | | | |
| **B)** Use an integrated approach | 1 | 2 | 3 | 4 | 5 | 6 | 7 | |
| **3. When I practised for the simulation throughout the semester I focused on:** | | | | | | | | |
| **A)** Just get the skills right | 1 | 2 | 3 | 4 | 5 | 6 | 7 | |
|  | | | | | | | | |
| **B)** Use an integrated approach | 1 | 2 | 3 | 4 | 5 | 6 | 7 | |
| **4. I found it easier to practice the simulation when I focused on:** | | | | | | | | |
| **A)** Just get the skills right | 1 | 2 | 3 | 4 | 5 | 6 | 7 | |
|  | | | | | | | | |
| **B)** Use an integrated approach | 1 | 2 | 3 | 4 | 5 | 6 | 7 | |
| **5. The simulation felt more real-life when I focused on:** | | | | | | | | |
| **A)** Just get the skills right | 1 | 2 | 3 | 4 | 5 | 6 | 7 | |
|  | | | | | | | | |
| **B)** Use an integrated  approach | 1 | 2 | 3 | 4 | 5 | 6 | 7 | |
| **6. Nearing the time that I was to do the simulation, I focused my preparation on:** | | | | | | | | |
| **A)** Just get the skills right | 1 | 2 | 3 | 4 | 5 | 6 | 7 | |
|  |  |  |  |  |  |  |  | |
| **B)** Use an integrated  approach | 1 | 2 | 3 | 4 | 5 | 6 | 7 | |

**7.** When did you get access to the information required for you to participate in the simulation?

**Please tick one of the following:**

Start of Semester ₁ Mid-Semester ₂ End of Semester ₃ No Access ₄

**8.** Was there time allocated to practise the simulation elements? **Please tick one of the following:**

Yes ₁ No ₂ ***If you answer No please go to Question 17.**

**9.** If you were given practice time; when were these practice sessions for the simulation scheduled? **Please tick one of the following:**

During the class ₁ Extra practice outside of class time ₂ Both during and after class ₃

**10.** Were there adequate practice periods/times for your simulation preparation? **Please tick one of the following:**

Not adequate ₁ Adequate ₂ More than adequate ₃

**11.** Did you use the practice sessions/times made available for your simulation? **Please tick one of the following:**

Not at all ₁ Sometimes ₂ Mostly ₃ Always ₄

**12.** When you attended the practice sessions/times did you receive comments and/or feedback from the teaching staff about your simulation performance? **Please tick one of the following:**

Not at all ₁ Sometimes ₂ Mostly ₃ Always ₄

**13.** How helpful did you find these comments and/or feedback about your simulation performance? **Please circle the number most applicable to you-**

| **1** | **2** | **3** | **4** | **5** | **6** | **7** |
| --- | --- | --- | --- | --- | --- | --- |
| Very  Unhelpful | Unhelpful | Slightly Unhelpful | Undecided | Slightly Helpful | Helpful | Very Helpful |

**14.** When you attended the practice sessions/times did you receive comments and/or feedback from someone other than the teaching staff, for example a fellow student about your simulation performance? **Please tick one of the following:**

Not at all ₁ Sometimes ₂ Mostly ₃ Always ₄

**15.** How helpful did you find these comments and/or feedback from others who were not teaching staff? **Please circle the number most applicable to you:**

| **1** | **2** | **3** | **4** | **5** | **6** | **7** |
| --- | --- | --- | --- | --- | --- | --- |
| Very  Unhelpful | Unhelpful | Slightly Unhelpful | Undecided | Slightly Helpful | Helpful | Very Helpful |

**16.** What did you practice at these sessions? **Please Tick the letter of your answer-**

**A)** focused only on the practical skills

**B)** the whole simulation but mainly focussed on the practical skills

**C)** focused on just the practical skills I struggle with, but also considered some aspects of the patient scenarios e.g. communication

**D)** the whole simulation and focusing on my patient using all aspects of the nurse-patient encounter

**17.** If you have any further comments you would like to add about the simulation please do so below:

Thank you very much for your time.
