# Supplementary material for: OSCE best practice guidelines—applicability for nursing simulations
Source: Adv Simul (Lond). 2016 Apr 2;1:10. doi: 10.1186/s41077-016-0014-1 (PMC5806284; doi:10.1186/s41077-016-0014-1)
Supplement: Supplementary file 2 — Examples of questions in students’ focus groups. (DOCX 24 kb) [file 41077_2016_14_MOESM2_ESM.docx]

**Appendix 2 – Examples of questions in students’ focus groups**

1) Why do you think your subject co-ordinator released the details of your simulation when they did?

2) How do you think the timing of release of the details of the simulation impacts upon your learning?

3) At UTS the simulation is timed toward the end of the course. Do you think this is appropriate to your learning? How does it help? How does it hinder?

4) In what ways did you seek comments and/or feedback at the simulation practice during your labs that you attended?

5) Can you explain how the comments and/or feedback affected your simulation performance?

6) What did you think were key points of focus for good performance in the simulation?

7) What is the value of the debriefing (facilitated debrief/reflection) session after your simulation?

8) How did the simulation prepare you for future clinical experience?
